# Supplementary material for: Embodiment modifies attention allotment for the benefit of dual task performance
Source: Commun Biol. 2022 Jul 14;5:701. doi: 10.1038/s42003-022-03603-6 (PMC9283402; doi:10.1038/s42003-022-03603-6)
Supplement: Supplementary file 2 — Description of Additional Supplementary Files [file 42003_2022_3603_MOESM2_ESM.pdf]

Description of Additional Supplementary Files

File name: Supplementary Data 1
Description: The source data behind the Figure 2 in the paper.

File name: Supplementary Data 2
Description: The source data behind the Figure 3 in the paper.

File name: Supplementary Data 3
Description: The source data behind the Figure 4 in the paper.

File name: Supplementary Data 4
Description: The source data behind the Figure 5 in the paper.
