# Supplementary material for: Embodiment modifies attention allotment for the benefit of dual task performance
Source: Commun Biol. 2022 Jul 14;5:701. doi: 10.1038/s42003-022-03603-6 (PMC9283402; doi:10.1038/s42003-022-03603-6)
Supplement: Supplementary file 7 — Reporting Summary [file 42003_2022_3603_MOESM7_ESM.pdf]

nature research

Corresponding author(s): Yukiko Iwasaki

Last updated by author(s): 27/4/22

Reporting Summary

Nature Research wishes to improve the reproducibility of the work that we publish. This form provides structure for consistency and transparency in reporting. For further information on Nature Research policies, see our Editorial Policies and the Editorial Policy Checklist.

Statistics

For all statistical analyses, confirm that the following items are present in the figure legend, table legend, main text, or Methods section.

n/a	Confirmed	
☐	☒	The exact sample size (*n*) for each experimental group/condition, given as a discrete number and unit of measurement
☐	☒	A statement on whether measurements were taken from distinct samples or whether the same sample was measured repeatedly
☐	☒	The statistical test(s) used AND whether they are one- or two-sided<br>*Only common tests should be described solely by name; describe more complex techniques in the Methods section.*
☐	☒	A description of all covariates tested
☐	☒	A description of any assumptions or corrections, such as tests of normality and adjustment for multiple comparisons
☐	☒	A full description of the statistical parameters including central tendency (e.g. means) or other basic estimates (e.g. regression coefficient) AND variation (e.g. standard deviation) or associated estimates of uncertainty (e.g. confidence intervals)
☐	☒	For null hypothesis testing, the test statistic (e.g. *F*, *t*, *r*) with confidence intervals, effect sizes, degrees of freedom and *P* value noted<br>*Give P values as exact values whenever suitable.*
☒	☐	For Bayesian analysis, information on the choice of priors and Markov chain Monte Carlo settings
☒	☐	For hierarchical and complex designs, identification of the appropriate level for tests and full reporting of outcomes
☐	☒	Estimates of effect sizes (e.g. Cohen's *d*, Pearson's *r*), indicating how they were calculated

Our web collection on statistics for biologists contains articles on many of the points above.

Software and code

Policy information about availability of computer code

Data collection	Unity
Data analysis	Matlab, R

For manuscripts utilizing custom algorithms or software that are central to the research but not yet described in published literature, software must be made available to editors and reviewers. We strongly encourage code deposition in a community repository (e.g. GitHub). See the Nature Research guidelines for submitting code & software for further information.

Data

Policy information about availability of data

All manuscripts must include a data availability statement. This statement should provide the following information, where applicable:

- Accession codes, unique identifiers, or web links for publicly available datasets
- A list of figures that have associated raw data
- A description of any restrictions on data availability

The final dataset used for the plots is provided as supplementary materials. Detailed datasets generated during and/or analyzed during the current study are available from the corresponding author on reasonable request.

Field-specific reporting

Please select the one below that is the best fit for your research. If you are not sure, read the appropriate sections before making your selection.

☐ Life sciences ☒ Behavioural & social sciences ☐ Ecological, evolutionary & environmental sciences

For a reference copy of the document with all sections, see nature.com/documents/nr-reporting-summary-flat.pdf

Behavioural & social sciences study design

All studies must disclose on these points even when the disclosure is negative.

Study description	quantitative experimental
Research sample	30 participants took part in this study (Mean age=25.5, SD=3.92, 23 males). Of these, 6 subjects who were first time VR users were removed after we observed that first time users were distracted and concentrated more on the left robot and task, rather than the main right hand task. The study was approved by the local ethics committee at the University of Montpellier, France as well as the ethics committee at Waseda University, Tokyo (13 participants were appended with additional experiments in Tokyo). All participants gave informed consent for their participation in the study. Overall the participant number of twenty four corresponded to our power analysis in G*Power 337 to provide 95% statistical power to achieve a medium effect size (d = 0.80) using a Wilcoxon signed rank test against an alpha of .05, and to provide 80% statistical power to achieve an effect size f= 0.2 in the main effect across conditions using an ANOVA repeated measures against an alpha of .05.
Sampling strategy	We used a student email mailing list to advertise the requirement of participants, and the participants were chosen on first come first serve basis.
Data collection	The experiment was in Virtual reality and the data was recorded directly by the computer during the experiments. Only the experimenter and participant was present during the experiment. The experimenter was not double blind to the conditions.
Timing	The data was collected from 04/02/2020 to 09/07/2021
Data exclusions	6 participants who were first time VR users were removed after we observed that first time users were distracted and concentrated more on the left robot and task, rather than the main right hand task.
Non-participation	none
Randomization	all participants performed both conditions in the study

Reporting for specific materials, systems and methods

We require information from authors about some types of materials, experimental systems and methods used in many studies. Here, indicate whether each material, system or method listed is relevant to your study. If you are not sure if a list item applies to your research, read the appropriate section before selecting a response.

Materials & experimental systems

n/a	Involved in the study	
☒	☐	Antibodies
☒	☐	Eukaryotic cell lines
☒	☐	Palaeontology and archaeology
☒	☐	Animals and other organisms
☐	☒	Human research participants
☒	☐	Clinical data
☒	☐	Dual use research of concern

Methods

n/a	Involved in the study	
☒	☐	ChIP-seq
☒	☐	Flow cytometry
☒	☐	MRI-based neuroimaging

Human research participants

Policy information about studies involving human research participants

Population characteristics	see above
Recruitment	see above
Ethics oversight	The study was approved (approved study ID 1911A) by the Ethical committee of Euromov, University o Montpellier.

Note that full information on the approval of the study protocol must also be provided in the manuscript.
